# Acute aortic regurgitation due to aortic valve leaflet injury during percutaneous coronary intervention: a case report

**DOI:** 10.3389/fcvm.2026.1806987

**Published:** 2026-06-12

**Authors:** Paweł Jańczak, Piotr Pawluczuk, Jakub Żmuda, Małgorzata Wojciechowska

**Affiliations:** 1 Chair and Department of Experimental and Clinical Physiology, Laboratory of Centre for Preclinical Research, Medical University of Warsaw, Warsaw, Poland; 2 Department of Invasive Cardiology, Independent Public Specialist Western Hospital John Paul II, Lazarski University, Grodzisk Mazowiecki, Poland

**Keywords:** echocardiography, heart team decision-making, iatrogenic valve injury, myocardial infarction, rotational atherectomy

## Abstract

Acute aortic regurgitation is a rare but potentially life-threatening complication of percutaneous coronary intervention (PCI). We report the case of a patient admitted with ST-segment elevation myocardial infarction who underwent primary PCI with rotational atherectomy due to heavily calcified coronary lesions. The procedure was initially successful; however, shortly after the intervention, the patient experienced sudden clinical deterioration with acute heart failure. Transthoracic and transesophageal echocardiography revealed an iatrogenic injury to the aortic valve leaflet, resulting in severe acute aortic regurgitation that contributed to the rapid hemodynamic deterioration. This case highlights a very rare but serious complication of PCI with rotational atherectomy. Aortic valve leaflet injury leading to acute aortic regurgitation should be considered in patients who develop sudden hemodynamic instability after complex coronary interventions. Early recognition with prompt echocardiographic assessment is crucial for appropriate management and may be life-saving.

## Case presentation

An 85-year-old patient was admitted with ST-segment elevation anterior myocardial infarction (STEMI) after 5 h of chest pain.

His past medical history included persistent atrial fibrillation, chronic heart failure with preserved ejection fraction (HFpEF), arterial hypertension, diabetes mellitus type II, and a previous ischemic stroke.

The patient had no history of coronary artery disease and had been hospitalized for HFpEF a week before at a remote hospital, where he underwent echocardiography as part of cardiological diagnostics. The examination revealed a preserved left ventricular ejection fraction, an enlarged left atrium, thickened left ventricular myocardium, mild atrioventricular valve regurgitation, and a normally appearing aortic valve.

**Figure 1:**
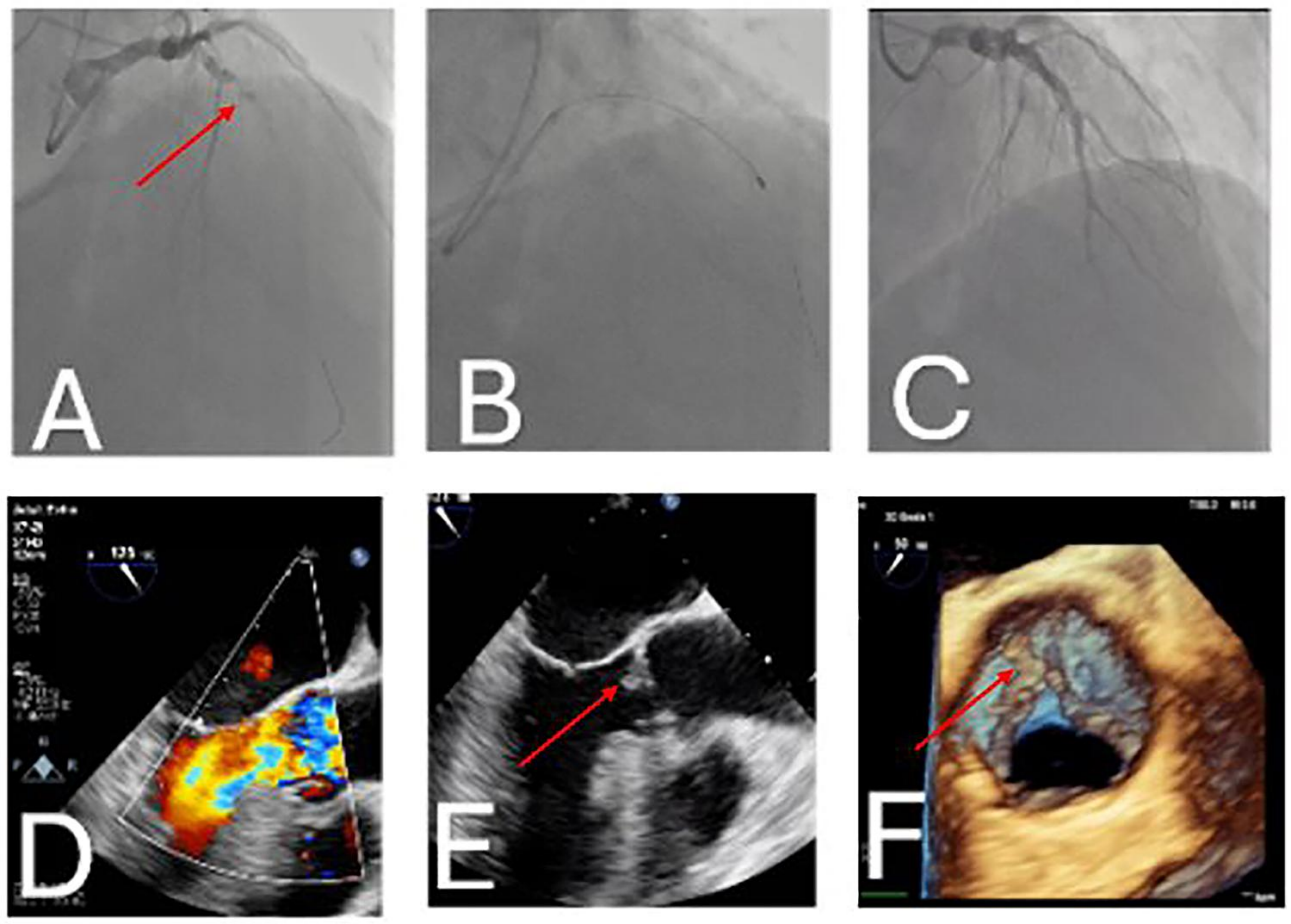
**(A)** Coronary angiography of the LAD indicating critical stenosis in the mid-portion of the vessel (arrow). **(B)** Rotablation of an uncrossable lesion in the LAD. **(C)** LAD after implantation of two DESs. **(D)** Transesophageal echocardiogram (TEE), color Doppler image showing severe aortic regurgitation. **(E)** TEE indicating a mobile structure on the non-coronary cusp (arrow). **(F)** TEE showing a 3D view of the injured non-coronary leaflet (arrow).

Upon admission, the patient presented with chest pain and no signs of heart failure. He was referred for urgent coronary angiography, which was performed using radial access with a JL 3.5, 6 Fr guiding catheter. The examination revealed single-vessel disease, namely, severe stenosis of the left anterior descending artery (LAD) with a tandem critical lesion, massive calcification in the mid-portion of the vessel, and Thrombolysis in Myocardial Infarction (TIMI) flow grade 1/2 (Figure 1A).

Because of an uncrossable lesion during primary percutaneous coronary intervention (PCI), a decision to perform rotablation was made (RotaLink Plus 1.5; 3 min 40 s ablation time in 15 s runs) (Figure 1B).

During the exchange from the rotawire to a workhorse wire following rotablation, ventricular fibrillation occurred. The patient was successfully defibrillated, and the PCI was subsequently completed without further complications. The vessel was predilated with non-compliant (NC) balloons; two drug-eluting stents (DESs) were implanted (3.0/22 and 3.5/30), with successful postdilatation with 3.5 NC balloons. TIMI flow grade 3 was achieved (Figure 1C). The patient was clinically stable, and his chest pain subsided.

However, over the following hours, the patient’s clinical condition deteriorated, with progression to acute Killip class IV left ventricular failure, requiring inotropes and vasopressor support and ultimately necessitating intubation and mechanical ventilation. Echocardiography showed an impaired ejection fraction (EF) of the left ventricle (35%) and features of severe aortic regurgitation, with an additional structure on a non-coronary aortic leaflet. Transesophageal echocardiography confirmed acute aortic regurgitation and non-coronary leaflet rupture (Figures 1D–F), with no aortic dissection. The patient experienced two episodes of cardiac arrest the next day, both due to asystole. He was resuscitated, and an endocavitary lead was placed.

Considering the patient had no history of fever, negative blood cultures, and low inflammation parameters, infective endocarditis was excluded.

The patient was evaluated by the Heart Team, and treatment options, including the possibility of urgent surgical or transcatheter aortic valve replacement (TAVI), were discussed; however, as these interventions are not available at our center and would have required transfer to a tertiary referral hospital, and in light of a Glasgow Coma Scale score of 4 with an extremely poor neurological prognosis, the patient was deemed not suitable for intervention. Pharmacological treatment in the ICU continued without success. The patient died on day 9 due to multiple organ failure.

## Discussion

Iatrogenic aortic valve injury during PCI is an extremely rare complication, with a prevalence of ≈1:10,000 (1). In the majority of cases, valve replacement and repair are life-saving procedures (2, 3). However, in our case, the patient was disqualified from such treatment. During the differential diagnosis, infective endocarditis and aortic dissection were excluded (2, 4).

A potential mechanism of valvular injury in this case may have involved the collapse of the guiding catheter into the contralateral aortic sinus, generating static mechanical stress, which, together with dynamic forces during rotational atherectomy and extensive catheter manipulation, could have contributed to valvular damage. Due to his elderly age, the patient’s aortic valve cusps may have become fat-infiltrated and degenerated, which could also have contributed to the trauma (5). Similar complications have been described in the literature, including cases related to Amplatz catheters (1, 2, 6).

### Rotational atherectomy in the setting of STEMI

Rotational atherectomy in the setting of STEMI remains a selective bailout strategy, primarily reserved for heavily calcified culprit lesions in which conventional PCI techniques fail to achieve adequate lesion crossing or optimal expansion. It is required in only a small proportion of STEMI cases and is driven by procedural necessity rather than planned use. Although STEMI has traditionally been regarded as a relative contraindication due to the risk of distal embolization and no-reflow, increasing patient age and the growing complexity of coronary anatomy have occasionally made plaque modification necessary to achieve procedural success (7). This is typically encountered in lesions characterized by dominant calcific stenosis rather than a high thrombus burden. With increasing operator experience in complex coronary interventions, the procedure can be performed safely in carefully selected patients (8). However, the current evidence base remains limited and is derived predominantly from observational studies.

In the present case, rotational atherectomy was not anticipated at the beginning of the procedure, and the initial guiding catheter (JL 3.5, 6 Fr) was selected according to the standard primary PCI strategy for STEMI. In acute settings, particularly in elderly patients undergoing transradial intervention, the use of a Judkins Left catheter for rapid engagement of the left coronary artery is generally considered reasonable.

Although a more supportive guiding catheter (e.g., Extra Backup or Amplatz) and a larger lumen (7 Fr) are typically preferred when rotational atherectomy is planned, the procedure in this case was continued using the initial setup, and guide exchange was avoided after procedural escalation to prevent further delay in reperfusion, which was considered critical in the acute setting.

### Treatment of acute severe iatrogenic aortic regurgitation

According to the available literature, acute severe aortic regurgitation due to mechanical valve injury is typically managed with urgent surgical intervention, including aortic valve replacement or, in selected cases, valve repair (2). Emerging data suggest that TAVI may represent a feasible alternative in selected high-risk or inoperable patients with pure aortic regurgitation (9). In the present case, however, such options were not feasible due to severe neurological injury and an extremely poor overall prognosis.

To the best of our knowledge, this is the first reported case of iatrogenic aortic valve injury caused during PCI that contributed to the patient’s death, which makes it an educational and unique case that should be carefully studied.

## Data Availability

The original contributions presented in the study are included in the article/Supplementary Material; further inquiries can be directed to the corresponding author.

## Supplementary material

Supplementary Video 1 Mid-esophageal long-axis transesophageal echocardiographic view of the aortic valve demonstrating severe aortic regurgitation.

Supplementary Video 2 Mid-esophageal short-axis transesophageal echocardiographic view of the aortic valve (3D TEE) showing an additional echogenic structure adjacent to the non-coronary cusp.

Supplementary Video 3 Mid-esophageal long-axis transesophageal echocardiographic view of the aortic valve showing an additional echogenic structure adjacent to the non-coronary cusp.

Supplementary Video 4 Rotational atherectomy of the left anterior descending artery (LAD).
